# The lived experience of adolescents with X-linked hypophosphataemia treated with burosumab at end of skeletal growth: a mixed-methods analysis

**DOI:** 10.1371/journal.pone.0344902

**Published:** 2026-03-20

**Authors:** Vrinda Saraff, Pedro Arango Sancho, Justine Bacchetta, Annemieke M. Boot, Christine Burren, Amish Chinoy, Poonam Dharmaraj, Maria Amelia Gómez Llorente, Juan David González Rodríguez, Iva Gueorguieva, Elin Haf Davies, Wesley Hayes, Sandra Komarzynski, Héctor Ríos Duro, Angela J. Rylands, Kerry Sandilands, Angela Williams, Emily Hardie, Haruka Ishii, Dirk Schnabel, Santhani M. Selveindran, Agnès Linglart

**Affiliations:** 1 Birmingham Women’s and Children’s Hospital, Birmingham, United Kingdom; 2 Sant Joan de Déu Barcelona Hospital, Barcelona, Spain; 3 Hospices Civils de Lyon, INSERM1033 Research Unit, Lyon, France; 4 University Medical Center Groningen, University of Groningen, The Netherlands; 5 University Hospitals Bristol and Weston NHS Foundation Trust, Bristol, United Kingdom; 6 Royal Manchester Children’s Hospital, Manchester, United Kingdom; 7 Alder Hey Children’s Hospital, Liverpool, United Kingdom; 8 Hospital Virgen de Las Nieves, Granada, Spain; 9 Department of Pediatric Nephrology, Santa Lucía General University Hospital, Cartagena, Spain; 10 Centre Hospitalier Universitaire de Lille, Lille, France; 11 Aparito Ltd, a wholly owned subsidiary of Eli Lilly and Company, Wrexham, United Kingdom; 12 Great Ormond Street Hospital, London, United Kingdom; 13 Pediatric Nephrology, Vall d’Hebron University Hospital, Barcelona, Spain; 14 Kyowa Kirin International, Marlow, United Kingdom; 15 Kyowa Kirin Co., Ltd., Tokyo, Japan; 16 Center for Chronically Sick Children, Pediatric Endocrinology, Charité – University Medicine Berlin, Germany; 17 Open Health Ltd, Marlow, United Kingdom; 18 Paris-Saclay University, AP-HP, INSERM, Service d’Endocrinologie et Diabète de l’Enfant, Bicêtre Paris-Saclay Hospital, Le Kremlin-Bicêtre, France; University of Padova: Universita degli Studi di Padova, ITALY

## Abstract

X-linked hypophosphataemia is a rare, genetic, lifelong disorder caused by phosphate-regulating endopeptidase homologue X-linked pathogenic variants and, if left untreated, is associated with a progressive accumulation of musculoskeletal manifestations. Burosumab is a fully human monoclonal antibody that targets circulating fibroblast growth factor 23 and directly inhibits its activity, thereby correcting the abnormal phosphate homoeostasis in people with X-linked hypophosphataemia (XLH). The efficacy and safety of burosumab has been demonstrated in a programme of clinical trials in children and adults. Few data describe the experience of adolescents with XLH receiving burosumab treatment before and after skeletal growth ends. This prospective, multicentre, mixed-methods study described the lived experience of adolescents with XLH treated with burosumab at the end of skeletal growth (NCT05181839). Using patient-reported outcomes, wearable devices, and interviews, we found low median symptom severity scores for pain (0.00), stiffness (0.00), and fatigue (1.75) on a 0–10 scale. Symptoms were usually triggered by physical activity but rarely interfered with daily life. Some adolescents reported emotional concerns related to XLH and treatment transition. These insights can inform patient support during transition to adult care.

## Introduction

X-linked hypophosphataemia (XLH) is a rare genetic disorder caused by loss-of-function mutations in the *PHEX* (phosphate-regulating endopeptidase homologue X-linked) gene. [1] Elevated fibroblast growth factor 23 (FGF23) reduces renal phosphate reabsorption and active vitamin D (1,25[OH]_2_D) production, resulting in chronic hypophosphataemia [1,2].

XLH typically becomes apparent in early childhood with rickets, skeletal deformities, short stature and dental abscesses [3,4]. Furthermore, short stature and skeletal deformities acquired in childhood are irreversible after completion of growth, and some require corrective limb surgeries [3,5]. Children also commonly experience impaired mobility and physical functioning, through delayed walking, abnormal gait, muscle weakness and painful bones, joints and muscles [5]. As hypophosphataemia persists into adulthood, the development of fractures, pseudofractures, enthesopathies, and early-onset osteoarthritis are common, and pain, stiffness and loss of physical function often accompany these [4,6]. Furthermore, children and adolescents suffer multiple emotional and social difficulties, such as lack of self-esteem or being self-conscious, and adults report mental health difficulties of anxiety and depression, and challenges with caregiving [6–9].

Treatment with multiple daily doses of oral phosphate supplements and active vitamin D is often poorly tolerated and burdensome for children and their caregivers, with variable efficacy and risks, including nephrocalcinosis and secondary hyperparathyroidism [3,10].

Burosumab, a fully human monoclonal antibody (immunoglobulin G1) targeting FGF23, has demonstrated efficacy and safety in children with XLH in multiple trials. Efficacy and safety of burosumab in children have been demonstrated in phase 2 and 3 trials [11–13]. A subgroup analysis of the UX023-CL201 phase 2 study further showed continued benefit of treatment with burosumab through adolescence [14].

Few data describe the experience of adolescents with XLH receiving burosumab treatment before and after skeletal growth ends. The end of skeletal growth (EoSG) refers to the cessation of height increase and the radiological fusion of growth plates, typically marking the completion of the skeletal growth phase [15]. The current observational, prospective, European, multicentre, mixed-methods study was designed to describe the lived experience of adolescents treated with burosumab at the EoSG (NCT05181839) [16]. The study extended over a period prior to and after the EoSG and included those who continued or discontinued burosumab after the EoSG. Here, we report the lived experience of adolescents treated with burosumab in a period prior to the EoSG.

## Methods

### Study design and setting

The study design has been published previously [16]. Briefly, the study utilised a Creswell Plano Clark convergent parallel design, whereby quantitative and qualitative data were collected and analysed simultaneously and independently, then compared [17].

Adolescents were recruited between 24 November 2021 and 19 October 2023 from 14 well-established specialist centres in the UK, France, Germany, Spain and The Netherlands that treated adolescents with XLH and had access to burosumab. The recruitment period differed for each site (**Table 1**).

**Table 1:** Recruitment period by study site.

Site	Country	Date first recruited	Date last recruited
Birmingham Women and Children’s Hospital	UK	24-Nov-21	13-Dec-22
Alder hey	UK	21-Feb-22	12-Jan-23
APHP Paris – Assistance Publique Hopitaux de Paris	FRA	25-Mar-22	29-Aug-22
Centre Hospitalier Universitair de Lille	FRA	4-Apr-22	11-May-22
Charité – Universitätsmedizin Berlin	GER	4-Apr-22	4-Apr-22
University Medical Centre Groningen – Beatrix Children’s Hospital	NTL	28-Jun-22	8-Dec-22
Bristol Royal	UK	16-Aug-22	16-Aug-22
Hosp. Gral Univ Santa Lucia	SPA	28-Sep-22	28-Sep-22
Manchester	UK	30-Nov-22	19-Oct-23
Hospices Civils de Lyon	FRA	16-Dec-22	16-Dec-22
Val d’Hebron	SPA	12-Dec-22	20-Jan-23
Great Ormond Street Hospital	UK	17-Jan-23	24-Jan-23
Hosp. S Joan de Deu	SPA	23-Jan-23	23-Jan-23

Note: Recruitment was initiated in one additional site in Spain (Virgen de las Nieves Maternidad Infantil); however, no participants were recruited.

FRA, France; GER, Germany; NTL, The Netherlands; SPA, Spain; UK, United Kingdom

Data collection concluded on 22 May 2024. The study involved two data collection periods close to the confirmed EoSG. The pre-EoSG period included 4 weeks of data collection within 12 weeks prior to the confirmed EoSG, and the post-EoSG period included 26 weeks of data collection, starting from the date of confirmed EoSG.

### Ethics statement

This study was conducted in accordance with the Declaration of Helsinki. Ethical approval was obtained from the institutional ethics committee at each participating centre as follows: The WEST II IEC – Angers, France Recherche et collections biologiques (Research and Biological Collections; RBC) identification No.: 2021-A01781-40, IEC identification no.: 2021/58, IS identification No.: 21.01296.000003; The Ethics Committee at the Charité Virchow Hospital Campus (Application number: EA2/237/22); the South West – Cornwall & Plymouth Research Ethics Committee & NHS Health Research Authority and Health and Care Research Wales (IRAS project ID:297382; REC reference:21/SW/0081); and the Fundació Sant Joan de Déu EC (EC code: EOM-29–21). Additionally, the Medical Ethics Review Board of the University Medical Center Groningen reviewed the protocol and determined that, according to the Medical Research Involving Human Subjects Act (WMO), formal approval was not required as the study did not constitute clinical research with human subjects as defined by the act. All participants provided written informed consent.

An amendment to the study protocol was implemented after initial ethics approval (Protocol version 2.0; 18 April 2023). This amendment included revising the sample size wording from “up to 30 adolescents” to “approximately 30 adolescents”; adding alternative units for data collection for parathyroid hormone (ng/L and pmol/L), alkaline phosphatase (IU/L), and serum phosphate (mg/dL); clarifying that source data verification would be conducted for 10% of the overall cohort (removing “at each centre”); and updating the planned study end date to October 2024. The amended protocol received ethics approval for all participating countries/sites, and the informed consent form was updated accordingly and re-signed by participants where applicable. Protocol deviations at the participant or site level (e.g., visit window deviations and consent-related deviations) were documented and managed in accordance with the study plans.

### Study population

Eligible participants were aged 12–17 years at the start of the study, with a confirmed diagnosis of XLH in their medical records and evidence of hypophosphataemia and/or impaired phosphate reabsorption due to elevated FGF23 and/or *PHEX* mutation.

Adolescents received burosumab for at least 12 months and were estimated to reach EoSG within the subsequent 26 weeks (based on treating clinical judgement in accordance with their normal approach in routine clinical practice). Adolescents who had missed more than one injection of burosumab in the previous 6 months or who were scheduled for orthopaedic surgery during the study period were not eligible. One participant was excluded from the analysis due to stopping burosumab treatment prior to the pre-EoSG data collection. The target sample size of 30 adolescents was considered appropriate and feasible for this mixed-methods study, which is not hypothesis-driven. Further details have been previously published [16].

### Data collection and variables

Prior to the study outset, in consultation with treating physicians, the sponsors engaged with four adolescents from the patient advocacy group ‘XLH UK’ to inform the study design and selection of outcome measures, and to assess the feasibility and acceptability of the proposed data collection method. Further details are published elsewhere [16].

Quantitative outcomes data were collected from medical records in MACRO Electronic Data Capture, and directly from the adolescent using a bespoke designed smartphone app (Atom5^TM^; Aparito, Wrexham, Wales, UK) to measure Worst Pain, Worst Fatigue, and Worst Stiffness, as well as an activity diary and health-related quality of life (HRQoL) using the EuroQol 5-Dimension Youth questionnaire (EQ-5D-Y), as described in **Table 2**, and paired with a wearable device (Garmin vívosmart^®^ 4; Garmin, Ltd., Olathe, KS, USA). The Garmin vívosmart^®^ 4 is a wrist-worn wearable device that collects minute-by-minute information on step count, heart rate (HR), and moderate-to-vigorous physical activity (MVPA), and was used to collect daily data over 4 weeks.

**Table 2:** Quantitative outcomes data collected during pre-EoSG data collection phase.

Source	Endpoint	Schedule during pre-EoSG phase (4 weeks)		
		Daily	Weekly at end of each week	Once at start of 4 weeks
Smartphone app	Worst pain, stiffness and fatigue in past 24 hours	X		
Smartphone app	Pain location and use of pain medication	X		
Smartphone app	Social and leisure activity diary		X	
Smartphone app	Time missed off school or work related to XLH		X	
Smartphone app	Healthcare resource use		X	
Smartphone app	HRQoL (EQ-5D-Y)			X
Wearable device (watch)	Step count (daily and hourly) and time in MVPA	X		

HRQoL, health-related quality of life; MVPA, moderate-to-vigorous physical activity; XLH, X-linked hypophosphataemia.

To collect qualitative data on the experiences of adolescents that would complement and expand understanding of the quantitative findings, 1:1 telephone interviews were performed using a semi-structured interview guide. Discussion topics included the experience of symptoms and their impact, emotional well-being, existing support networks and hopes for the future. All interviews were audio-recorded and carried out by trained qualitative researchers.

### Data analysis

Data were analysed descriptively and included all adolescents who met the inclusion criteria and were receiving burosumab treatment during pre-EoSG data collection phase. XLH-related medical and treatment history, height and local laboratory data are presented by sex. The World Health Organization reference values were used to calculate height z-scores [18]. No imputation for missing data was considered.

Laboratory values were obtained by local laboratories as part of routine clinical care.

Patient-reported symptom severity was assessed using a 0–10 scale (10 being the worst possible intensity) for each symptom: pain, fatigue, stiffness. A total symptom score out of 30 was calculated by combining individual symptom scores. Weekly and 28-day median scores were calculated for each symptom, and cohort medians were derived from individual medians. Correlations between symptoms were assessed using the Spearman correlation coefficient, with a significance level of *P* < .05 (two-tailed). Within-adolescent (individual-level) changes and between-adolescent differences (cohort-level) were assessed across the 4 weeks. The difference in individual patient highest and lowest weekly median symptom scores across the 4 weeks were also calculated.

The pain scores of adolescents who took pain medication at least once were compared with the pain scores of those who never took pain medication. For adolescents who took pain medication, the median pain scores on days where pain medication was taken were calculated and compared descriptively with the median pain scores on days where pain medication was not taken.

The daily wear time of the Garmin vívosmart^®^ 4 was assessed by inspecting for missing HR data at the time of analysis. Epochs greater than or equal to 5 minutes with missing HR and null values for steps and physical activity intensities were classified as non-wear. The total step count per day and step count taken by hour (i.e., hourly step count) were computed from the minute-by-minute steps data. The time spent in MVPA was derived from the physical activity intensities recorded by the device and presented as minutes per day. MVPA was calculated using the Garmin vívosmart® 4’s algorithm, which classifies physical activity intensity by comparing real-time HR data to the user’s average resting HR [19]. In the absence of HR data, the device estimates moderate intensity based on steps per minute [19]. Daytime was defined as between 05:00 and 23:00, with valid days requiring at least 7 hours of wear time; the wear time analyses were restricted to these same waking hours, wearing the device at night was optional. Weekly medians (interquartile range [IQR]) for each adolescent were calculated using a threshold of at least 3 days’ data in the week. These individual weekly median scores were then used to calculate a weekly median score for the full cohort. A 28-day median score was also calculated using all data available for each patient. The 28-day median score for the full cohort was calculated as the median of the individual 28-day median scores.

EQ-5D-Y data were presented as frequencies of responses at item level, median (IQR) EQ visual analogue scale (VAS), and median (IQR) EQ-5D-Y index values. EQ-5D-Y index values were calculated using the Spanish value set for each patient and R package ‘eq5d’ [20]. Statistical analyses were carried out using R version 4.2.2.

Qualitative data from interviews were analysed using the Framework approach to identify themes across different groups of adolescents, as well as trajectories within individual adolescents [21].

Activities diaries were rated by five authors (AW, AJR, EH, KS, VS) as low-, medium- or high-energy activities. The final rating was according to the majority.

## Results

### Adolescent characteristics and treatment history

Twenty-five adolescents consented and participated in the study, but one was excluded from this pre-EoSG analysis, as they had stopped burosumab at the beginning of data collection; therefore, this analysis considers the remaining 24 adolescents.

The characteristics and treatment history of the adolescents are reported in **Table 3**. Fifteen adolescents (63%) were female. The median (IQR) age at XLH diagnosis was 3.1 (1.3, 4.7) years for females and 2.5 (0.2, 2.6) years for males, and the median (IQR) age of treatment initiation with burosumab was 11.9 (10.8, 12.6) years for females and 13.2 (9.3, 13.8) years for males. Eleven adolescents (45.8%) started treatment with burosumab at age > 12 years.

**Table 3:** Adolescent characteristics and treatment history.

Characteristic	Overall	Female (n = 15)	Male (n = 9)
**Age at study start,** years			
Median (IQR)	15.0 (14.0, 16.2)	15.0 (14.0, 15.5)	16.0 (16.0, 17.0)
**Height standard deviation score**			
Median (IQR)	–0.8 (−1.7, −0.1)	–0.7 (−1.6, −0.4)	–1.1 (−1.7, 0.0)
Weight,* kg			
Median (IQR)	54.0 (49.5, 64.1)	51.0 (46.5, 55.5)	64.1 (56.5, 69.2)
**Body Mass Index (BMI),** kg/m^2^			
Median (IQR)	21.8 (20.2, 23.0)	21.8 (20.1, 22.9)	21.8 (20.4, 27.9)
**Age at XLH diagnosis,** years			
Median (IQR)	2.6 (1.0, 3.9)	3.1 (1.3, 4.7)	2.5 (0.2, 2.6)
**Age at burosumab initiation,** years			
Median (IQR)	11.9 (10.6, 12.9)	11.9 (10.8, 12.6)	13.2 (9.3, 13.8)
**Age at EoSG,** years			
Median (IQR)	16.4 (15.4, 17.5)	15.6 (15.0, 16.5)	17.7 (16.8, 17.9)
**Age at burosumab initiation,** years, n (%)			
**≤12**	13 (54.2)	9 (60.0)	4 (44.4)
**>12**	11 (45.8)	6 (40.0)	5 (55.6)
**Years of exposure to burosumab up to EoSG,** n (%)			
1–2	3 (12.5)	2 (13.3)	1 (11.1)
>2–3	3 (12.5)	1 (6.7)	2 (22.2)
>3–4	5 (20.8)	4 (26.7)	1 (11.1)
> 4–5	7 (29.2)	6 (40.0)	1 (11.1)
>5	6 (25.0)	2 (13.3)	4 (44.4)
**EoSG assessment**, n (%)			
Growth velocity	10 (41.7)	9 (60.0)	1 (11.1)
Imaging	4 (16.7)	1 (6.7)	3 (33.3)
Growth velocity and imaging	4 (16.7)	2 (13.3)	2 (22.2)
Other	2 (8.3)	2 (13.3)	0
Growth velocity and imaging and Tanner staging and	2 (8.3)	1 (6.7)	1 (11.1)
other			
Imaging and other	1 (4.2)	0	1 (11.1)
Withdrawal before EoSG	1 (4.2)	0	1 (11.1)

*Weight missing for one male patient.

EoSG, end of skeletal growth; IQR, interquartile range; XLH, X-linked hypophosphataemia.

Females were treated with burosumab prior to EoSG for a median (IQR) of 4.1 (3.6, 4.6) years, whereas males were treated for 4.5 (3.0, 8.1) years. The median (IQR) age at the EoSG was 15.6 (15.0, 16.5) years for females and 17.7 (16.8, 17.9) years for males. At EoSG, the median (IQR) height z-score was –0.7 (−1.6, −0.4) for females and –1.1 (−1.7, 0.0) for males.

### Laboratory values

Laboratory values measured within 6 months of the EoSG were available for 17 adolescents (71%). The median (IQR) values for serum phosphate, alkaline phosphatase and parathyroid hormone were 0.9 (0.8, 1.0) mmol/L, 142.0 (121.0, 204.0) U/L, and 5.3 (4.5, 6.7) pmol/L, respectively. Based on local reference ranges, the number of adolescents within normal range for serum phosphate, alkaline phosphatase and parathyroid hormone were 10 (58.8%), 9 (52.9%), and 12 (70.6%), respectively.

### Symptoms

**Fig 1:**
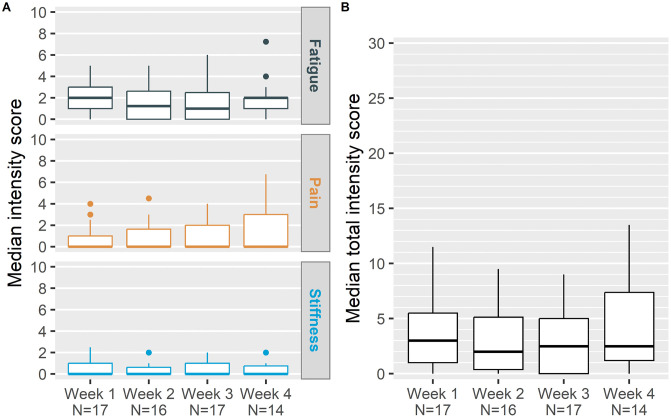
Symptom intensity scores reported by adolescents with XLH during the 4-week pre-EoSG period. (A) Median intensity score for Worst Fatigue (top panel), Worst Pain (middle panel), and Worst Stiffness (bottom panel); (B) Median total symptom intensity scores calculated as the sum of individual symptom scores. Sample sizes (N) varied by week and are indicated below each timepoint. EoSG, end of skeletal growth; IQR, interquartile range; N, number of patients.

The median (IQR) number of days in the symptom diary completed by the 24 adolescents was 13.0 (9.5, 22.0). The 28-day median (IQR)–scores for worst daily fatigue, pain and stiffness in the previous 24 hours were 1.8 (0.4, 3.0), 0.0 (0.0, 1.6), and 0.0 (0.0, 1.0), respectively. The 28-day median (IQR) total symptom score (combined fatigue, pain, and stiffness score) was 3.8 (1.6, 5.1). During the 4-week data collection period, stiffness scores had the least variation across the full cohort, with slightly more between-patient variation in fatigue and pain scores (Fig 1**).**

Individual adolescent weekly median scores for stiffness and fatigue were the most consistent across the 4 weeks, with 17 (94.44%) having less than a 2-point difference in weekly median score. Individual patient weekly median pain scores varied slightly more, with a greater than 2-point change in five (27.78%) adolescents (**Table 4**) [22]. There were weak correlations between pain and fatigue scores (ρ = 0.23, *P* = 0.286) and pain and stiffness scores (ρ = 0.38, *P* = 0.066), although not statistically significant, and there was no correlation between fatigue and stiffness (ρ = 0.06, *P* = 0.763).

**Table 4:** Distribution of patients by change in weekly median symptom scores (0–10 NRS) over the 4-week pre-EoSG period.

Range of change (points)	0	>0–1	>1–2	>2–3	>3–4
Fatigue	6	5	6	1	0
Pain	8	1	4	3	2
Stiffness	11	3	3	1	0

EoSG, end of skeletal growth; NRS, numerical rating scale

### Fatigue

Interviews revealed that nearly half (45.8%) of participants experienced fatigue; this was daily for some (n = 3) and several times a week for others (n = 2). Of those who reported fatigue, the majority (n = 10) described feeling this after physical activity (e.g., walking, physical education, gym). One adolescent explained: “When I do activities, if it’s an activity which requires a lot of movement, for example, physical education and that, um, yes, I sometimes get tired.” Another adolescent said that he/she experienced fatigue after school: “The tiredness comes after being in high school. After I get home, I need some time to rest and stand up and do other things.”

Three adolescents stated that the fatigue lasted more than 30 minutes.

Six adolescents said that their fatigue did not interfere with any activities; however, four said that it interfered with their education such that they would sometimes have to miss school, miss or be unable to participate in physical education/sports lessons at school, or had difficulties completing schoolwork.

Three adolescents expressed that the fatigue affected them emotionally and negatively, with one saying: “So, it’s feeling like crying a lot all the time. Besides, I’m a little low emotionally, so, it all comes together.” Furthermore, the interference of fatigue left them feeling ‘frustrated’, ‘not good’, ‘annoyed’, upset’ and that this was ‘unfortunate.’

### Pain

Pain was reported at least once by 18 adolescents (75.0%), of whom nine (50.0%) used pain medication at least once on days with pain (**Table 5**). The median (IQR) pain intensity score among adolescents who used pain medication was higher than in those who did not (3.0 [2.0, 4.0] vs 2.0 [1.1, 4.8]) and was higher on the days when they took pain medication compared with the days that they did not (median [IQR] intensity: 3.0 [3.0, 6.0] vs 2.0 [2.0, 3.0]).

**Table 5:** Pain location and medication use in the pre-EoSG period.

	Adolescents (n = 24)
**Reporting pain at least once**, n (%)	18 (75.0)
**Used pain medication at least once**, n (%)	9 (50.0)
**Reporting pain location at least once**, n (%)	
Head	6 (33.3)
Chest	0
Spine	9 (50.0)
Shoulder	2 (11.1)
Upper arm	1 (5.6)
Lower arm	0
Elbow	1 (5.6)
Wrist and hand	1 (5.6)
Fingers	1 (5.6)
Upper leg	6 (33.3)
Lower leg	9 (50.0)
Knee	12 (66.7)
Ankle	8 (44.4)
Foot	4 (22.2)

EoSG, end of skeletal growth

Pain was also reported during interviews, with 17 adolescents (70.8%) experiencing pain, most commonly (n = 13) with physical activities. The most common areas of pain were the knee (12; 66.7%), lower limbs (9; 50.0%), spine (9; 50.0%), and ankle (8; 44.4%), based on the symptom diaries (**Table 5**). Duration of the physical activity also impacted pain occurrence for some adolescents (n = 6): “It’s only if I’ve been walking for a really long time. But maybe like… 30, 40 minutes walking… maybe I then notice that your legs just really hurt.”

Pain was often described as achy (n = 9), with other descriptions including sore/bruise-like, stretched muscle, muscle fatigue, nagging pain, heavy/pressure, ‘knees can’t hold me up’, mismatched joints, stabbing pain, and ‘growing pains.’

Most adolescents (n = 8) felt the pain was minimal, manageable and not severe. Most adolescents (n = 10) reported that pain did not interfere with their daily activities, although five adolescents reported interference with physical activities.

Most adolescents (n = 11) did not report being emotionally affected by the pain; two adolescents explained that this was because the pain was minimal or that they were accustomed to having pain and nothing could be done about it. Three adolescents reported feeling negative emotions when the pain was more severe, including sadness, anger and irritation.

Three adolescents who reported pain explained that the pain had been more severe before starting burosumab. Four adolescents who did not report pain mentioned experiencing it before starting burosumab, and that pain usually started due to activity. One adolescent stated:

*Well, I personally… then, I don’t know if it is the case for everybody but I know that before…before burosumab, during the last year or two before changing to burosumab, for example when I was going to classes, given the “collège” [school] had different floors, for example I had trouble going up the stairs… so… there wasn’t just the stairs, it is just an example but, for example, when I ran, I couldn’t run, it hurt too much on my legs.*

### Stiffness

Stiffness was reported by 12 adolescents (50%) during interviews. Six were affected after periods of immobility (e.g., waking up in the morning, sitting or lying down for too long) and five after physical activity (e.g., gym, work, walking up hills, physical education) or at the end of the day. One adolescent commented, “Yeah, and stiffness, the morning after football, that’s… it’s obviously going to be like the most stiff as your muscles and everything....”

All adolescents who reported stiffness felt it in their backs, knees, ankles or legs; the occurrence and duration of stiffness varied across individuals. Stiffness was said to have occurred daily (n = 2), once or twice a week (n = 3), and twice a month (n = 1). The duration of stiffness ranged from a few minutes to 1–2 hours for some (n = 4), but could even last ‘all day’ (n = 1).

Stiffness was typically described in relation to moving; descriptions included ‘difficult to move’, ‘unable to move’, ‘cannot move in other direction’, or ‘a bit slow.’ However, most adolescents who had stiffness reported that it did not cause any or much interference with their daily living (n = 8). One adolescent explained, “It’s typically because it’s so early in the morning and towards the end of the day, I’m not usually doing much, so it doesn’t impact many of my activities.”

Two adolescents reported interference with activities; one stated, “If I’m at work the next day, I’ll struggle to lift the boxes that I probably wouldn’t usually struggle to lift…” Most adolescents (n = 6) did not feel emotionally impacted by their stiffness. However, three reported feeling annoyed, frustrated, and angry, and two reported a negative emotional impact from their stiffness interfering with daily life. One adolescent narrated, “Well, frustrated and also angry, because I say, I can, but this doesn’t let me. I mean, I have the strength to do it, but it’s as if my body is saying no, and so, I can’t.” Three adolescents who did not report stiffness mentioned experiencing stiffness before starting burosumab.

### Physical function and activity

**Fig 2:**
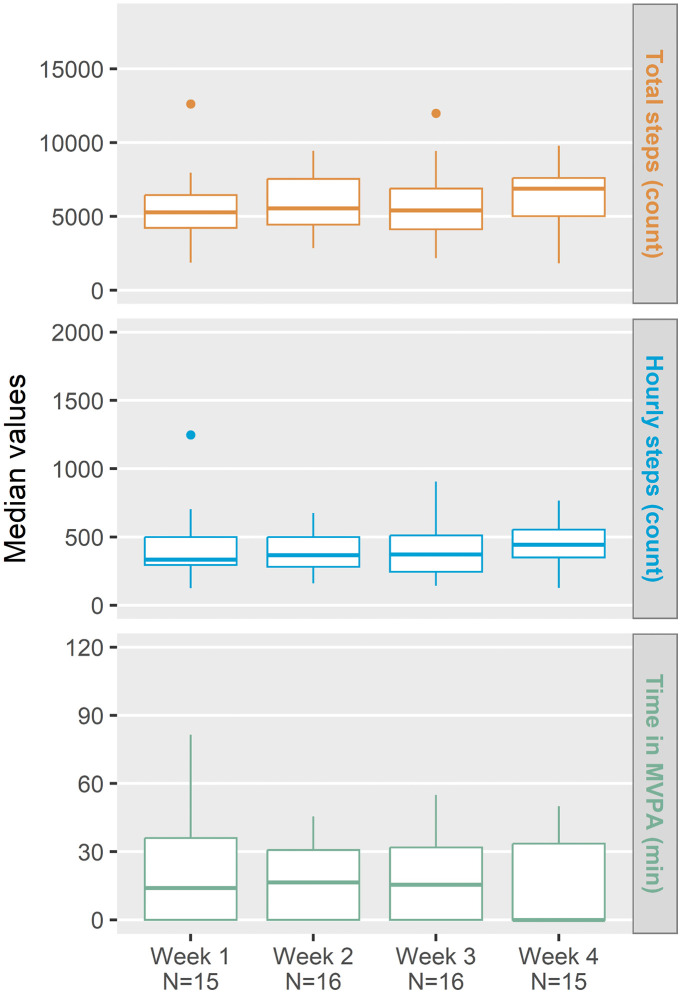
Weekly median data from wearable device during the 4-week pre-EoSG period. Panels show (from top to bottom): total daily steps (count), hourly steps (count), time spent in moderate-to-vigorous physical activity (MVPA, minutes); N reflects the number of adolescents with valid wearable data for each respective week. EoSG, end of skeletal growth; MVPA, moderate-to-vigorous physical activity.

Overall, 378 readings were available out of a maximum total of 637 (59.3%) from the wearable device. Twenty-two adolescents had a median (IQR) wear time of 14.7 (11.0, 16.8) hours during the defined daily wear time. Median (IQR) total daily step count varied between males (6852 [4041, 8225]) and females (5470 [4861, 7106]). Similarly, median (IQR) hourly step count was higher in males (544 [307, 664]) than in females (396 [316, 512]). The MVPA minutes per day was also higher in males, with a median (IQR) of 37.0 (12.3, 46.5) minutes compared with that in females of 10.0 (0.0, 24.3) minutes. Finally, there was little within-adolescent variation for each of the wearable metrics, but some variation between adolescents (Fig 2).

Nineteen adolescents (79.2%) completed the Typical School Term Activity Diary and 18 (75.0%) the Typical Holiday Activity Diary. Of the 43 different activities listed across the 4 weeks by the adolescents, 18 (41.9%) were rated as low-energy, 10 (23.3%) as medium-energy, and 15 (34.9%) rated as high-energy (**Table 6**).

**Table 6:** Activity diary.

Low-energy activities	Medium-energy activities	High-energy activities
Dressmaking	Work	Athletics
School/college	Cadets	Boxing
Wind band	Golf	Climbing
Shopping	Housework	Cycling
Cinema	Trip to beach	Dancing
Disney	Trip to fair	Football
Exams/studying/revision	Trip to town	Gym
Homework	Dog walk	Mixed sports
Music	Walking	Rugby
Resting	Frisbee	Running
Seeing family		Sport
Seeing friends		Working out
Travelling by car		Physical education
Video games		Baseball
Watching football		Swimming
Going out with friends		
Outing		
Driving lesson		

### Health-related quality of life

The EQ-5D-Y was completed by 20 adolescents (83%) — 12 females and eight males. Most respondents (≥90%) had no problems with mobility, looking after themselves, or doing their usual activities (**Table 7**); six adolescents (30%) reported feeling ‘some’ pain; and seven (35%) reported feeling ‘a bit’ or ‘very’ worried, sad or unhappy. Male adolescents rated their overall well-being slightly higher than females on the EQ-5D-Y VAS; the median (IQR) values were 87.50 (79.50, 91.25) vs 80.00 (61.3, 90.50). However, median (IQR) EQ-5D-Y index values were comparable across sexes 1.00 (0.91, 1.00) for males and 0.96 (0.71, 1.00) for females. The mean (standard deviation) index values were 0.96 (0.05) for males and 0.82 (0.25) for females.

**Table 7:** EQ-5D-Y scores in the pre-EoSG period.

	Adolescents (n = 20)
**EQ-5D-Y dimension**	
**Mobility**, n (%)	
No problems	18 (90.0)
Some problems	2 (10.0)
A lot of problems	0
**Looking after myself**, n (%)	
No problems	18 (90.0)
Some problems	2 (10.0)
A lot of problems	0
**Doing usual activities**, n (%)	
No problems	20 (100.0)
Some problems	0
A lot of problems	0
**Having pain or discomfort**, n (%)	
No problems	14 (70.0)
Some problems	6 (30.0)
A lot of problems	0
**Feeling worried, sad or unhappy**, n (%)	
No problems	13 (65.0)
Some problems	5 (25.0)
A lot of problems	2 (10.0)
**EQ-5D-Y index score,** median (IQR)	1.00 (0.89, 1.00)
**EQ-5D-Y VAS score,** median (IQR)	85.00 (73.75, 90.50)

EoSG, end of skeletal growth; VAS, visual analogue scale.

During interviews, most adolescents (n = 17) reported they were not sad or worried due to XLH because they ‘felt normal’ because the disease was not visible, symptoms and impact on daily life were minimal, and they had good support networks and psychological coping strategies such as stoicism and acceptance.

Those who reported feeling sad and worried did so due to the impact of the disease, symptoms, and treatment/change in treatment on their future career, family, daily activities, physical activities or travel plans.

There were worries related to future careers:

*Actually, it’s about the job I would like to do, I would like to work in law enforcement, and sometimes I am worried that it would prevent me from doing that...I’m scared that later, I will experience more pain, etc. and that it would prevent me from doing this.*

Initiation of a future family was a concern:

*I’ve been told that if I have kids, then the girl is 100% going to get [XLH] if I have a girl, which yeah, it’s… it’s not very nice thinking that you’re going to be the reason why a child should have so many doctors’ appointments or not be able to do certain things or not be as tall as they might want to be. It’s quite a lot…*

The trajectory of XLH was upsetting:

*My mum’s had quite a lot of operations on her legs due to XLH and, in the nicest possible way, I don’t want to end up like that. I don’t want them operations having to be done on me. Just because she can’t walk without sticks now, yeah.*

### Healthcare interactions and absenteeism

Over the 4-week period, six adolescents reported 10 routine appointments and no emergency appointments; these were categorised as specialist doctor visits (n = 4), other (n = 4), and dentist (n = 2).

In the interviews, 11 adolescents (45.8%) provided positive feedback on their healthcare providers (e.g., doctors, nurses, dentists), using words such as ‘good’, ‘perfect’, and that their healthcare providers were caring and communicated well. One adolescent reported: “*If I needed to see the doctor or see them within a week, they’ve been really good. There’s nothing I can complain about, everyone’s done everything they can to help me.”* The adolescents typically had appointments with their main XLH physician quarterly or twice yearly. Seven (29.1%) mentioned seeing dentists or surgeons yearly or twice yearly, and visits from the nurse every two weeks to receive the burosumab injections, and two reported missing some work or school due to XLH (1 full-day, 2 half-days).

#### Transition of care after EoSG, and the future.

Many of the adolescents (n = 13 [54.2%]) had not discussed transitioning to adult care with their treating physician. Some (n = 4 [36.3%]) who had the conversation with their doctor were still unclear on the details of transitioning:

*But I’m… I don’t really understand what happens from here, because I’m going to be turning 18 in March, and they’ll gauge whether they take me into adult care or not… I don’t know what that means to be honest. I know it’s a very different building, but yeah and then they were saying the adult care’s not as good, they’re not as bothered.*

Adolescent responses to transitioning to adult care were mixed:

*Uh, it makes me feel for one, a bit grown up but…not really any emotion, in particular, I think I’d miss the actual hospital itself ’cos I have a lot of good…well, as good as it can be considering I’m in a hospital, experiences there.*

When asked about their future, most (n = 21 [87.5%]) talked about doing well in their schoolwork, passing exams and going to university or joining apprenticeship programmes. Among those with social or recreational plans (n = 10 [41.2%]), three (30.0%) wanted to travel, six (60.0%) wanted to participate more in physical activities, and two (20.0%) talked about spending more time with friends. Finally, of the three (12.5%) adolescents asked about relationship goals, their main goal was centred on having a life partner, getting married and having a family.

## Discussion

Limited data describe the lived experience of adolescent patients with XLH, which is a pivotal phase of life as healthcare is transitioned from paediatric to adult services. Existing literature supports substantial burden and reduced health-related quality of life, which results from the accumulation of unresolved childhood disease complications, such as severe pain, stiffness and decreased physical activity [23].

In this study, adolescents with XLH receiving burosumab treatment for a median of 4 years reported minimal pain, stiffness and fatigue in a daily symptom diary at EoSG, suggesting a positive outcome of burosumab treatment in real-world clinical practice. The duration of treatment with burosumab may provide an explanation as to why the height of this cohort is not as compromised as might otherwise be expected. Of note, this interpretation is observational, and individual growth trajectories may vary.

Although symptom scores varied between individuals, fatigue and stiffness were generally stable across weeks. Pain was less stable within each adolescent, with more than 25% reporting a > 2-point difference in median weekly scores, and which is important to consider if adapting pain management. The lack of a strong correlation between symptoms suggests that pain, stiffness and fatigue are largely independent of one another and clinicians should inquire about each separately when discussing with their patients.

Although low, the highest symptom intensity was reported for fatigue, with more than half of adolescents reporting this during interviews; furthermore, this was reported after physical activity, corroborating literature that links excessive fatigue with physical exertion in 60%−67% of children and adolescents with XLH [7].

Most adolescents reported pain, and most commonly in lower limbs with physical activity. It was often described as ‘achy’, and half of all adolescents reporting pain also used pain medication at least once during the data collection period. In other literature, pain was also reported frequently in children and adolescents with XLH who were not receiving burosumab (28%−34%), and triggers included physical activity [7]. The reported prevalence of bone and joint pain has also been high in children (80%), and muscle pain has been reported in 60% of children [3]. While the lowest intensity scores were reported for stiffness, during interviews, approximately half reported stiffness, often after physical activity or inactivity. This aligns with a previous study in which stiffness was experienced by 38% of children with XLH, the majority of whom were not receiving burosumab [3].

To the authors’ knowledge, this study is the first to assess wearable data in adolescents with XLH. The daily step count in these adolescents was slightly lower than the 8000–9000 steps/day expected in healthy adolescents [24], and time spent in MVPA was lower than the recommended >60 minutes/day in healthy adolescents (41.54 minutes [males]; 24.39 minutes [females]) [25]. Daily activity diaries indicated that adolescents participated in activities of varying energy expenditure. Additionally, symptoms of fatigue, stiffness and pain were often due to physical activity but did not discourage on in such activities. Thus, physicians should be aware of symptom onset with physical activity and propose adapted options to ensure comfort for adolescents.

Our findings support previous literature that highlights that XLH in adolescence is multifactorial, with an increasing emotional burden characterised by anxiety, lack of confidence, low self-esteem and worries around starting a family, among other factors [8]. More than one-third of adolescents reported ‘some’ or ‘a lot’ of feeling worried, sad or unhappy. When explored during interviews, those who felt sad or worried described it as due to reasons such as the impact of the disease, symptoms and treatment, and the impact of changing treatment. Thus, it is important to address both the emotional and physical aspects of their health, through a multidisciplinary care approach.

The higher EQ-5D-Y mean index and VAS scores for adolescents in the study indicated better health-related quality of life than previously reported in a similar age group with XLH, in which patients were treated with oral phosphate supplements and active vitamin D, in which the mean EQ-5D-3L (and EQ-5D-3L proxy) index was 0.788 ± 0.153 and the mean VAS score was 68.33 ± 16.06 [20]. Furthermore, adolescents in this study had comparable scores to the English general population norms for corresponding age-groups [26].

This study provides a comprehensive evaluation of the experience of adolescents living with XLH through both quantitative and qualitative data collection. This provides a disease overview for physicians and equips multidisciplinary teams with in-depth insights that can guide supportive conversations with adolescents as they approach adult care. Although physical function is often measured in clinical settings through validated questionnaires and tests, these measures are limited to measuring a patient’s physical activity level as part of daily living. A wearable device can offer unique insight by enabling remote prolonged measurement of activity parameters [27].

Sex differences in physical activity were not specifically accounted for in this analysis [28]. Previous literature has shown that gender can influence physical activity levels, with adolescent males often engaging in higher levels of physical activity than females. Future research could explore gender differences in physical activity and their potential impact on clinical outcomes, particularly in relation to the effects of treatments such as burosumab [28].

Within the study, the clinical approach to assessing EoSG varied between sites and adolescents; although primarily assessed using growth velocity measures, there was no clear pattern and a combination of various methods was often used to determine the point of skeletal growth cessation. Furthermore, a 2-point cut off was used to assess change in weekly median scores, based on findings from the Brief Pain Inventory tool, this was considered appropriate due to the similarity in questions relating to worst pain and same scale being used [22]. As a non-interventional, real-world study conducted across multiple European centres in a rare disease setting, data collection was limited to parameters routinely measured and recorded by clinicians as part of standard clinical practice. As such, the sample size was limited, some degree of missing data was unavoidable, and some adolescents did not have a laboratory test performed within 6 months prior to EoSG. Furthermore, while BMI was derived from clinical records and is reported in Table 3, other potentially relevant variables, including prior therapy and functional tests of muscular performance, were not systematically collected as part of the study, although it is acknowledged that these can be valuable for evaluation. Variability in EoSG assessment methods across sites may limit comparability.

## Conclusions

This mixed-methods study is the first to explore the lived experience of adolescents with XLH at EoSG using both quantitative data from patient-reported outcomes and wearable devices enriched with qualitative data from interviews. After a median of 4 years of burosumab treatment, participants reported low pain, stiffness and fatigue, remaining physically active but occasionally affected emotionally—particularly regarding transition to adult care.
